# Impact of gut-peripheral nervous system axis on the development of diabetic neuropathy

**DOI:** 10.1590/0074-02760220197

**Published:** 2023-03-20

**Authors:** Thalita Mázala-de-Oliveira, Yago Amigo Pinho Jannini de Sá, Vinicius de Frias Carvalho

**Affiliations:** 1 Fundação Oswaldo Cruz-Fiocruz, Instituto Oswaldo Cruz, Rio de Janeiro, RJ, Brasil; 2 Cedars-Sinai Medical Center, Medicine Department, Los Angeles, CA, United States of America; 3 Instituto Nacional de Ciência e Tecnologia em Neuroimunomodulação, Rio de Janeiro, RJ, Brasil

**Keywords:** diabetes, dysbiosis, gut microbiota, inflammation, neuropathy

## Abstract

Diabetes is a chronic metabolic disease caused by a reduction in the production and/or action of insulin, with consequent development of hyperglycemia. Diabetic patients, especially those who develop neuropathy, presented dysbiosis, with an increase in the proportion of pathogenic bacteria and a decrease in the butyrate-producing bacteria. Due to this dysbiosis, diabetic patients presented a weakness of the intestinal permeability barrier and high bacterial product translocation to the bloodstream, in parallel to a high circulating levels of pro-inflammatory cytokines such as TNF-α. In this context, we propose here that dysbiosis-induced increased systemic levels of bacterial products, like lipopolysaccharide (LPS), leads to an increase in the production of pro-inflammatory cytokines, including TNF-α, by Schwann cells and spinal cord of diabetics, being crucial for the development of neuropathy.

Diabetes is a chronic metabolic disease characterized by hyperglycemia due to a reduction in the production and/or action of insulin.^1^ Currently, diabetes is one of the most serious and frequent chronic diseases in worldwide. Uncontrolled diabetes is accompanied by the development of several disabling and costly complications, which reduce patients’ life expectancy and can be fatal.^2^ In 2021, the global prevalence of diabetes reached pandemic proportions with 537 million people living with diabetes in the world, accompanied by an expense of 699 billion USD in global healthcare. In addition, future projections suggest that up to 2045 the number of people with diabetes will increase by 46%, with an estimate of health expenditures for the care of this disease that will exceed one trillion USD.^3^

Neuropathy is the most prevalent complication of diabetes, occurring in up to half of all people living with this disease.^4^ In addition, neuropathy is responsible for frequent hospitalization compared to other diabetes morbidities,^5^ and it is the most common reason for non-traumatic amputation.^6^ Neuropathic pain is manifested as spontaneous or induced pain, such as hyperalgesia and allodynia.^7^ ^,^ ^8^ Although neuropathy is the strongest predictor of mortality in diabetes, it remains without specific treatment.^4^ This scenario leads to high individual costs for patients, including pain, inability to work, poor quality of life, multiple hospitalizations for ulcers and eventual amputations. Therefore, we performed a narrative review with the aim of increasing knowledge about the role of gut dysbiosis in the development and/or progression of neuropathy in diabetic patients, besides how essential this microenvironment is for better control of the disease.

Pathogenesis of diabetic neuropathy

In diabetic patients, the development of neuropathy is multifactorial and involves uncontrolled glycemia, diabetes duration, and age-related neuronal attrition.^4^ ^,^ ^6^ Although the precise order of cellular injury in diabetes is unknown, the alterations in the peripheral nervous system that culminate in diabetic neuropathy are well established. These changes include (i) progressive loss of neurofilament polymer, which are essential structural scaffolds of the axon;^9^ (ii) modification in the key plasticity molecules in the dorsal root ganglia (DRG), including decrease in the synthesis of growth-associated protein 43 (GAP43) and β-tubulin and increase in the expression of heat shock proteins (HSP) and poly(ADP-ribose) polymerase (PARP);^10^ ^,^ ^11^ ^,^ ^12^ ^,^ ^13^ (iii) axonal degeneration;^14^ (iv) reduction in the Schwann cells to support axons, through decrease in the provision of cytoskeletal support, trophic factors or ribosome transfer that allows intra-axonal mRNA translation within distal axons;^15^ (v) demyelination, which occurs in more severe cases of diabetic neuropathy;^16^ ^,^ ^17^ ^,^ ^18^ (vi) reduction in the blood flow in the DRG^19^ (Fig. 1).

**Fig. 1:**
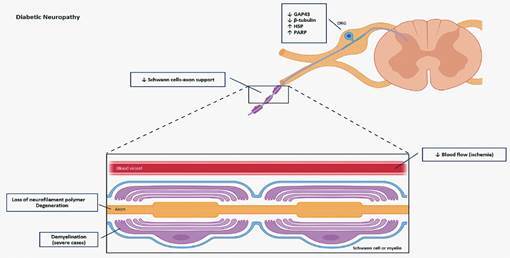
alterations observed in the peripheral nervous system in diabetic neuropathy. In the dorsal root ganglion (DRG), localization of the cell bodies of sensory neurons, diabetes induced alterations in the key plasticity molecules, including reduction in growth-associated protein 43 (GAP43) and β-tubulin in parallel with an increase in the heat shock proteins (HSP) and poly(ADP-ribose) polymerase (PARP) expression. Furthermore, diabetics showed loss of neurofilament polymer, decrease in the Schwann cells-axon support, axonal degeneration, and ischemia in the sensory nerves, in addition to a demyelination in more severe cases of diabetic neuropathy. Altogether, these alterations evoked pain in diabetic patients.

The exact mechanism that promotes alterations in the peripheral nervous system and, consequently, neuropathic pain in diabetic patients is not fully elucidated. Nonetheless, several evidences showed that hyperglycemia and dyslipidemia induce pathological changes in neurons, glia, and vascular cells that culminate in nerve dysfunction and neuropathy.^4^ Hyperglycemia increases glycolysis, polyol, advanced glycation end products, protein kinase C, and hexosamine pathways in Schwann cells, DRG neurons and axons.^16^ ^,^ ^17^ ^,^ ^18^ ^,^ ^20^ These metabolic changes result in increased reactive oxygen species (ROS) formation and release of pro-inflammatory mediators. In parallel, the dyslipidemia observed in diabetic patients also induced a rise in ROS production and systemic and local inflammation.^21^ ^,^ ^22^

In diabetes, the overproduction of ROS induces activation of PARP-1 with a concomitant decrease in ATP formation.^23^ Altogether, high levels of ROS and loss of ATP production cause mitochondrial failure and metabolic and oxidative damage in Schwann cells and DRG neurons.^24^ ^,^ ^25^ The increase in ROS formation, dysfunctional mitochondria, and microvascular insufficiency result in axonal degeneration in the peripheral nervous system.^26^ ^,^ ^27^ ^,^ ^28^ ^,^ ^29^ In addition, the oxidative damage induces hyperexcitability in the axons and DRG neurons, causing neuropathic pain.^30^

Several pre-clinical and clinical studies showed upregulation of pathways involved in inflammation in peripheral nerves.^31^ ^,^ ^32^ ^,^ ^33^ ^,^ ^34^ Furthermore, numerous experimental models of diabetic neuropathy showed an inflammatory response, characterized by infiltration of macrophages and T cells and increased levels of pro-inflammatory cytokines, in the sciatic nerve and DRG.^35^ ^,^ ^36^ ^,^ ^37^ ^,^ ^38^ The inflammatory response was accompanied by loss of myelinated and unmyelinated nerve fibers and axonal damage in diabetic animals with neuropathy.^39^ The presence of inflammation biomarkers was also associated with onset and progression of neuropathy in diabetic patients.^40^ ^,^ ^41^ Moreover, diabetes-induced inflammation altered mitochondrial bioenergetics in DRG neurons^42^ ^,^ ^43^ and HSP content in sensory neurons.^44^ ^,^ ^45^ Besides, the systemic low-grade inflammation has been implicated in neuropathic pain observed in diabetic patients, since some circulating inflammatory mediators was positive related to the severity of neuropathic pain in a subgroup of patients with distal symmetrical polyneuropathy.^46^

Diabetes and gut microbiota

The human gut microbiota contains up to 100 trillion of microbes, including commensal, symbiotic, and pathogenic bacteria, as well as archaea, fungi, and viruses.^47^ ^,^ ^48^ Gut microbiota has various physiological functions in the host, including strengthening epithelial-intestinal barrier integrity,^49^ maintenance of energy homeostasis,^50^ protection against pathogens,^51^ and regulation of host immunity.^52^

Diet, early-life microbiota exposure, antibiotic therapy, changing hygiene status, pollution, socioeconomic status, and other environmental factors can directly influence the composition of gut microbiota and its metabolic products, making it unique to each individua.^53^ ^,^ ^54^ Furthermore, patients with some pathologic conditions, for instance inflammatory diseases, infections, neurodegenerative diseases, and metabolic diseases, presented an imbalance in the microbes’ composition into the gut with the predominance of pathogenic bacteria, known as dysbiosis.^55^ ^,^ ^56^

In early stages of life, the extensive exposure to antibiotics may lead to dysbiosis, resulting in underweight or overweight.^57^ ^,^ ^58^ ^,^ ^59^ Moreover, the large consumption of antibiotics is usually been related to the development of metabolic disorders in later stages of life.^60^ In addition, the intake of sweeteners, as sugar substitute, also has considerable impact over microbiota population, inducing glucose intolerance.^61^ ^,^ ^62^ These observations suggest that an imbalance in the composition of the gut microbiota may be related to the development of metabolic diseases. In fact, the composition of gut microbiota can alter the immune response of non-obese diabetic (NOD) mice, a classical model of autoimmune diabetes. Germ-free (GF) NOD mice showed an acceleration in the insulitis in parallel to a rise in the Th1 and Th17 cells in the mesenteric and pancreatic lymph nodes,^63^ however, the incidence of diabetes was not modified.^63^ ^,^ ^64^ Furthermore, the administration of probiotics in NOD mice decreased the incidence of diabetes, through reduction of insulitis and gut permeability and modulation of cytokine profile, Treg cells and T helper cell polarization.^65^ ^,^ ^66^ ^,^ ^67^ These data indicate that gut microbiota is important to the development of diabetes.

It is well known that diabetic patients showed dysbiosis.^68^ ^,^ ^69^ Although the composition of gut microbiota is diverse among diabetic patients, the relationship between *Firmicutes* and *Bacteroidetes* is unbalanced in these patients.^70^ ^,^ ^71^ ^,^ ^72^ In addition, patients with type 1 diabetes presented a clear depletion of species like *Prevotella copri* and *Bifidobacterium longum*, probiotic bacteria, and enrichment of families like *Ruminococcaceae*, *Clostridiaceae*, *Clostridiales*, and *Oscillibacter*, bacteria associated with infection and inflammation.^73^

The maintenance of homeostasis in gut environment is important not only to slow-down diabetes development but it is also central in the control of its complications.^74^ Dysbiosis in diabetic patients and animals lead to an increase in the intestinal permeability^75^ ^,^ ^76^ in parallel to a rise in the bacterial content to the bloodstream, as lipopolysaccharide (LPS).^68^ ^,^ ^77^ Interestingly, the supplementation of diabetic mice with a microbial anti-inflammatory molecule, which is a metabolite of a commensal bacteria *Faecalibacterium prausnitzii*, improved the intestinal barrier permeability and reduced circulating levels of LPS.^78^

In diabetic patients, the gut leakiness is accompanied by a low-grade inflammation, with increased levels of IL-1β, IL-6, and TNF-α in the blood.^79^ Furthermore, type 1 and type 2 diabetic patients as well as NOD mice showed a reduction in the abundance of butyrate-producing bacteria.^80^ ^,^ ^81^ Butyrate is a short-chain fatty acid (SCFA) that induce mucin production, regulating the permeability of the intestinal barrier, and reduce translocation of bacteria and their products, oxidative stress, and inflammation.^82^ The reduction of butyrate-forming bacteria, with consequent inadequate butyrate secretion, aggravates the pathogenesis of diabetes,^83^ through increase of inflammation and oxidative stress. Treatment with butyrate decreased ROS production and the homeostatic levels of inflammatory markers in diabetic mice.^84^

Remarkably, treatment with probiotics is one of the most used strategies to modulate intestinal microbiota and their used can prevent diabetes establishment and is effective as adjuvant in insulin resistance therapies.^85^ ^,^ ^86^ ^,^ ^87^ ^,^ ^65^ ^,^ ^66^ ^,^ ^67^ According to Food and Agriculture Organization (FAO) and World Health Organization (WHO), probiotics are defined as live micro-organisms which when administered in adequate amounts confer a health benefit on the host.^88^ Furthermore, treatment of diabetic rats with probiotics slow down the progression of diabetes in clear association with a decrease in the plasma levels of LPS.^89^ Besides, the treatment of diabetic animals with probiotics containing the *Lactobacillus rhamnosus* NCDC17 improved the insulin resistance, in parallel to a reduction in IL-6 and TNF in the epididymal fat.^90^ Although the use of adjuvant therapy with probiotics seems to be interesting to treat some comorbidities of diabetes related to dysbiosis, some effects of them are controversy in diabetic patients. In general, the treatment with probiotics is useful to control insulin resistance and improved the intestinal barrier permeability in type 2 diabetic patients, however, it is ineffective in reducing systemic inflammation. Furthermore, the use of probiotics to reduce the glycemia and serum insulin levels in these patients is very controversy. Although the studies with type 1 diabetic patients are still scarce, it was observed that treatment with probiotics reduced glycemia and systemic inflammatory markers as compared to placebo (Table).

**TABLE:** Clinical trials in diabetic patients after probiotic administration

Prob	Study design/subjects	Sample size	Study period	Advantage	Disadvantage	Ref
*Lactobacillus acidophilus* NCFM^®^ (10^10^ CFU)	Double-blinded, randomized, placebo-controlled, T2D males. Ages: 48-65 years-old	Prob: *n =* 21 Cont: *n =* 24	4 weeks	↑ insulin sensitivity	No alteration in the inflammatory markers (TNF, IL-6, IL-1ra and CRP)	^91^
*Lactobacillus acidophilus* (2 ×10^9^CFU); *L. casei* (7×10^9^ CFU); *L. rhamnosus* (1.5×10^9^ CFU); *L. bulgaricus* (2×10^8^ CFU); *Bifidobacterium breve* (2×10^10^ CFU); *B. longum* (7×10^9^ CFU), *Streptococcus thermophilus* (1.5×10^9^CFU)	Randomized, double-blinded, placebo-controlled, T2D males and females. Ages: 35-70 years-old	Prob: n = 27 Cont: n = 27	8 weeks	↓ FPG and CRP	No alteration in the serum insulin levels and HOMA-IR	^92^
*Lactobacillus sporogenes* (1×10^7^ CFU) + 0.04 g inulin (as prebiotic)	Randomized, placebo-controlled, T2D. Ages: 35-70 years-old	Prob: n = 35 Cont: n = 35	6 weeks	↓ Insulin and CRP	No alteration in the HOMA-IR and FPG	^93^
*Lactobacillus acidophilus* (4x10^8^ CFU/100mL); *Bifidobacterium bifidum* (4x10^8^ CFU/100mL)	Randomized, double-blind, placebo-controlled, T2D females. Ages: 50-60 years-old	Prob: n = 10 Cont: n = 10	5.5 weeks	↓ FPG and Glycemia	---	^94^
*Lactobacillus acidophilus* La-5 (10^9^ CFU); *Bifidobacterium animalis subsp lacti*s BB-12 (10^9^ CFU)	Randomized, double-blind, placebo-controlled, T2D patients. Ages: 35-60 years-old	Prob: n = 23 Cont: n = 22	6 weeks	↓ HbA1c and TNF- α (both groups)	No alteration in the IL-10 and IL-6 levels	^95^
*Lactobacillus casei* (4×10^10^ CFU)	Randomized, placebo-controlled, T2D patients. Ages: 30-79 years-old	Prob: n = 34 Cont: n = 34	16 weeks	↑ Counts of the total *Lactobacillus* and *L. casei* subgroup ↑ Counts of the total *Clostridium coccoides* group and the *C. leptum* subgroup ↓ *L. gasseri* and *L. reuteri* subgroups ↓ Translocation of gut bacteria to the blood	No alteration in the HbA1c, FPG, and inflammatory markers (LBP, IL-6, TNF-α, CRP)	^96^
*Lactobacillus acidophilus* La5 (7.23x10^6^ CFU/g); *Bifidobacterium lactis* Bb12 (6.04x10^6^ CFU/g)	Randomized, double-blind, controlled clinical, T2DM. Ages: 30-60 years-old	Prob: n = 30 Cont: n = 30	6 weeks	↓ FPG and HbA1c	No alteration in the insulin levels	^97^
*Lacticaseibacillus paracasei* (3x10^8^ CFU) strain Shirota (previously *Lactobacillus casei* strain Shirota); *Bifidobacterium breve* (3x10^8^ CFU) strain Yakult, and *galactooligosaccharides*)	Randomized, double-blind, controlled clinical, T2DM and obese patients. Ages: 30-80 years-old	Prob: n = 44 Cont: n = 42	24 weeks	↑ *B. adolescentis*, *B. pseudocatenulatum*, *Lactobacillus* ↓ *B. vulgatus*	↑ levels of FPG and HbA1c (12 weeks) No alteration in the glycemia and inflammatory markers (IL-6, LBP, CRP)	^98^
*Lactobacillus acidophilus* (2×10^9^ CFU); *Lactobacillus casei* (7×10^9^ CFU); *Lactobacillus rhamnosus* (1.5×10^9^ CFU); *Lactobacillus bulgaricus* (2×10^8^ CFU); *Bifidobacterium breve* (3×10^10^ CFU); *Bifidobacterium longum* (7×10^9^ CFU); *Streptococcus thermophilus* (1.5×10^9^ CFU)	Randomized, double-blind, controlled clinical, T2DM. Ages: 30-75 years-old	Prob: n = 30 Cont: n = 30	6 weeks	↓ FPG	No alteration in the insulin levels	^99^
*Lactobacillus casei* (10^8^ CFU/L)	Block randomized, controlled clinical, T2DM. Ages: 30-50 years-old	Prob: n = 20 Cont: n = 20	8 weeks	↓ FPG, serum insulin level, and HOMA-IR	No alteration in the HbA1c	^100^
*Lactobacillus + Lactococcus* (6×10^10^ CFU/g); *Bifidobacterium* (1×10^10^ CFU /g); *Propionibacterium* (3×10^10^ CFU/g); *Acetobacter* (1×10^6^ CFU /g)	Randomized, double-blind, controlled clinical, T2D. Ages: 18-75 years-old	Prob: n = 31 Cont: n = 22	8 weeks	↓ HbA1c and HOMA-IR	↑ TNF-α, IL-1β, IL-6, INF- γ levels No alteration in the FPG and serum insulin and IL-8 levels	^101^
*Lactobacillus salivarius* (subsp. *salicinius* AP-32); *Lactobacillus johnsonii* (MH-68); *Bifidobacterium animalis* (subsp. *lactis* CP-9) contain 1×10^10^ CFU/day)	Randomized, double-blind, placebo-controlled trial. T1DM. Ages: 6-18 years-old	Prob: n = 27 Cont: n = 29	24 weeks	↑ *B. animalis*, *Akkermansia muciniphila* and *Lactobacillus salivarius* in the gut ↓ FPG, HbA1c, IL-8, IL-17, MIP-1β, TNF-α ↑ TGF-β	---	^102^

CFU: colony-forming units; Cont: control; CRP: C-reactive protein; FPG: fasting plasma glucose; HbA1c: hemoglobin A_1_c; HOMA-IR: homeostasis model of assessment-insulin resistance; IL: interleukin; IL-1ra: interleukin-1 receptor antagonist; INF: interferon; LBP: lipopolysaccharide binding protein; MIP: macrophage inflammatory proteins; Prob: probiotic; TGF: transforming growth factor; TNF: tumor necrosis factor; T1D: type 1 diabetes; T2D: type 2 diabetes.

Gut microbiota and neuropathic pain

The gut microbiota plays a role in the maintenance of nervous system function, through immunological, hormonal, and neuronal signals.^103^ ^,^ ^104^ Several studies showed the participation of gut microbiota in the development of pain, since GF mice exhibited visceral hypersensitivity that was controlled by postnatal colonization with conventional microbiota.^105^ ^,^ ^106^ ^,^ ^107^ ^,^ ^108^ In addition, the transference of fecal microbiota from irritable bowel syndrome patients to GF mice increased visceral hypersensitivity.^109^ Likewise, probiotic treatment improves stress- and inflammation-induced visceral hypersensitivity.^110^ ^,^ ^111^ ^,^ ^112^ ^,^ ^113^ In addition, gut dysbiosis has been described in patients with irritable bowel syndrome who present abdominal pain.^114^ ^,^ ^115^ ^,^ ^116^ ^,^ ^117^

Furthermore, the gut microbiota is also involved in the pathophysiology of neuropathic pain. For instance, some chemotherapy drugs, including paclitaxel and oxaliplatin, cause chemotherapy-induced peripheral pain (CIPN) during anti-cancer treatment,^118^ and affects up to 48% of patients undergoing chemotherapy.^119^ Several chemotherapy drugs that induced neuropathic pain change the composition of gut microbiota, inducing dysbiosis.^120^ Likewise, oxaliplatin-induced mechanical hyperalgesia is decreased in GF mice or in animals treated with antibiotics,^121^ suggesting that CIPN depends on dysbiosis. Another case of gut microbiota participating in neuropathic pain is observed in a murine model of chronic constriction injury (CCI).^104^ In this model, the oral treatment with antibiotics resulted in gut microbiota changes and decreased the development of CCI-induced neuropathic pain, through a skewing from a pro-inflammatory to an anti-inflammatory immune profile,^122^ indicating that dysbiosis is an important factor in the development of CCI-induced neuropathic pain. In addition, the transplantation of fecal microbiota from rats with spared nerve injury to pseudo-GF mice increased mechanical stimulus-induced pain,^123^ suggesting that gut microbiota possess a significant role in the spared nerve injury-induced neuropathic pain.

Since the development of neuropathic pain can be related to changes in the gut microbiota and diabetic patients presented dysbiosis in association with neuropathy, a central question arises: can dysbiosis and the consequent pro-inflammatory status be critical for the development of neuropathic pain in diabetes?

Dysbiosis is important to the establishment and aggravation of diabetic neuropathy

In diabetes, dysbiosis occurs in parallel to a break in the epithelial-intestinal barrier and translocation of bacterial contents to the bloodstream.^68^ In addition, the hyperglycemia observed in diabetic patients is followed by an increase in the pro-inflammatory cytokines IL-1β, IL-6, and TNF-α, marking low-grade inflammation.^79^ Furthermore, the reduction in the butyrate-producing bacteria in the gut microbiota of diabetic patients is related to the high permeability of the epithelial-intestinal barrier.^124^

Interestingly, the incubation of Schwann cells in a high glucose environment induced an increase in the apoptosis of those cells in association with overexpression of TLR4, the receptor activated by LPS, and a rise in the TNF-α production.^125^ In addition, the expression of TLR4 mRNA and the protein levels TNF-α were increased in the spinal cord of streptozotocin-induced diabetes. These raises in TLR4 and TNF-α were positively correlated with mechanical/thermal hypersensitivity in diabetic rats.^126^ Reinforcing the hypothesis that the increase in TLR4 expression in the Schwann cells and spinal cord is important to diabetic neuropathy, the inhibition of TLR4 signaling in the spinal cord attenuated mechanical hyperalgesia in diabetic rats with neuropathy and downregulated the local levels of TNF-α.^127^ Furthermore, the continuous delivery of IL-10 in the nerve fibers of DRG blocked the nociceptive response in diabetic animals, in parallel to a decrease in the expression of TLR4.^128^

TNF-α is produced primarily by endoneurial macrophages and Schwann cells,^129^ and is increased at the injury site after CCI of the sciatic nerve in rats.^130^ ^,^ ^131^ The administration of TNF-α inhibitor or antibodies to TNF-α reduced nerve injury- and CCI-induced hypersensitivity, respectively.^132^ ^,^ ^133^ Furthermore, the IL1R1/TNFR1 double knock-out mice showed a decrease in the nociceptive sensitivity after nerve injury compared to wild-type littermates.^134^ In addition, direct injection of TNF-α into the sciatic nerve induced painful neuropathy.^135^ ^,^ ^136^ It is well known that patients with diabetic peripheral neuropathy showed elevated levels of both TNF-α and soluble TNF-α receptors in their serum.^137^ ^,^ ^138^ ^,^ ^139^ The inhibition of TNF-α, using a recombinant human TNF-α receptor-antibody fusion protein, recovery lower nerve conduction velocity, demyelination of nerve fibers, disorganization of lamellar and axonal structures, and decreased expression of myelin basic protein in the nerve tissue of diabetic rats that developed peripheral neuropathy.^140^ Furthermore, the blockage of TNF-α signaling using either TNF-α knockout mice or anti-TNF-α monoclonal antibody improved neuropathy in diabetic mice.^141^

Sodium butyrate has anti-inflammatory and neuroprotective effects in spinal cord injury, including the improvement of motor function and the reduction of neutrophils accumulation and pro-inflammatory cytokine expression.^142^ Furthermore, sodium butyrate and sodium propionate improved nitroglycerin-induced pain attacks, reducing the damage in the trigeminal nerve nucleus and the expression of pro-inflammatory mediators.^143^ Likewise, the pain and discomfort in healthy human were reduced after intraluminal administration of butyrate into the distal colon.^144^ Altogether, these evidences suggested that the decrease in the butyrate-producing bacteria in diabetic patients can be related to the development of neuropathic pain.

The composition of the gut microbiota can change during the development of diabetes. Interestingly, the diversity of gut microbiota from patients with type 2 diabetes with gastrointestinal autonomic neuropathy was modified compared to type 2 diabetic patients without this condition. Diabetic patients with neuropathy presented an increase in the relative abundance of pathogenic bacteria of phyla Proteobacteria,^145^ a LPS-producing bacteria phylum.^146^ Jinmaitong, a natural compound rich in flavonoid and its glycosides, triterpenoids, and phenolic acids,^147^ improves nerve conduction velocity, pain, and temperature sensation in diabetic rats with neuropathy,^148^ ^,^ ^149^ as well as markedly ameliorated clinical symptoms of pain in the extremities of diabetic patients with peripheral neuropathy.^150^ In parallel, Jinmaitong enriched nine species of gut microbiota of diabetic rats with neuropathy, avoiding dysbiosis.^151^

Furthermore, the flavonoid quercetin reversed mechanical pain and intraepidermal nerve fiber density in streptozotocin-induced diabetic rats, in clear association with the reduction of pathogenic bacteria species and the enrichment of two prebiotic species.^152^ These data suggest that both Jinmaitong and quercetin improve neuropathy in diabetic subjects by modulating phenotype-associated gut microbiota. In agreement with the proposition that dysbiosis can be important to the development of neuropathy in diabetes, a case report showed that fecal microbiota transplantation decrease limb pain and paresthesia in a diabetic patient with neuropathy that did not use any painkillers or drugs for alleviating the pain. In addition, this patient showed an improvement of motor conduction velocity in tibial nerve, attested by electromyogram, and a reduction in the visual analogue scale pain score from severe pain to mild pain after the treatment with fecal microbiota transplantation.^153^

In conclusion

In conclusion, we postulate that the increase in the richness of pathogenic bacteria and a reduction in the abundance of butyrate-producing bacteria in the gut microbiota of diabetic patients may be responsible for the onset of peripheral neuropathy. The dysbiosis in diabetes triggers a break in the intestinal barrier with consequent increase in the bacterial products, such as LPS, into the bloodstream. Possibly, the activation of TLR4 in the Schwann cells and spinal cord of diabetics induces an overproduction of TNF-α, resulting in the increase of pain (Fig. 2). In this respect, new therapeutic strategies founded on probiotics or bacterial metabolites, as butyrate, seem to be potentially practical approaches for adjuvant treatment of neuropathy in diabetic patients.

**Fig. 2:**
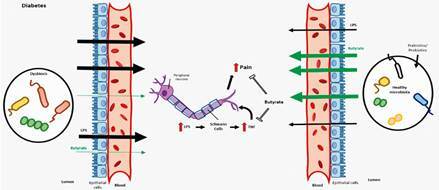
mechanisms associated with diabetic neuropathy induced by gut microbiota dysbiosis and the therapeutic effects of prebiotics/probiotics. Dysbiosis in diabetic patients results in a leak of epithelial-intestinal barrier, increasing its permeability and the absorption of molecules from lumen to the blood such as lipopolysaccharide (LPS). LPS directly activates Schwann cells to release tumor necrosis factor-α (TNF-α), favoring the nociception in diabetics. Changes in diet, prebiotics or probiotics may induces changes in microbiota, restoring butyrate producing microorganisms, leading to anti-inflammatory effects, reducing the TNF-α production and, consequently, the pain in diabetics tumor necrosis factor.
